# Multivariate analysis of TU wave complex on electrocardiogram in Andersen–Tawil syndrome with *KCNJ2* mutations

**DOI:** 10.1111/anec.12721

**Published:** 2019-11-14

**Authors:** Hitoshi Horigome, Yasuhiro Ishikawa, Norito Kokubun, Masao Yoshinaga, Naokata Sumitomo, Lisheng Lin, Yoshiaki Kato, Yuri Tanabe‐Kameda, Seiko Ohno, Masami Nagashima, Minoru Horie

**Affiliations:** ^1^ Department of Child Health Faculty of Medicine University of Tsukuba Tsukuba Japan; ^2^ Ishikawa Clinic Saitama Japan; ^3^ Department of Neurology Dokkyo Medical University Tochigi Japan; ^4^ Department of Pediatrics National Hospital Organization Kagoshima Medical Center Kagoshima Japan; ^5^ Department of Pediatric Cardiology Saitama Medical University International Medical Center Hidaka Japan; ^6^ Department of Bioscience and Genetics National Cerebral and Cardiovascular Center Suita Japan; ^7^ Aichi Saiseikai Rehabilitation Hospital Nagoya Japan; ^8^ Department of Cardiovascular and Respiratory Medicine Shiga University of Medical Science Otsu Japan

**Keywords:** Andersen–Tawil syndrome, electrocardiogram, independent component analysis, repolarization, U wave

## Abstract

**Background:**

The exact differences between the TU wave complex of ATS1 and that of healthy individuals remain to be investigated. We sought to characterize the TU wave complex of Andersen–Tawil syndrome type 1 (ATS1) using high frequency electrocardiogram (ECG) data.

**Methods:**

Electrocardiograms were recorded as time series data with a 2 kHz frequency ECG amplifier in 13 patients with ATS1 (positive for *KCNJ2* mutation, ATS1 group) and age‐matched healthy individuals (control group). Conventional ECG parameters were measured, and principal component analysis (PCA) and independent component analysis (ICA) were applied to the TU wave complex.

**Results:**

Time from T peak (Tp) to U peak (Up), time from bottom (B) to Up, and time from B to U end (BUe, U duration) (0.232 ± 0.018 vs. 0.165 ± 0.017, *p* < .0001), where B is the lowest point between T and U waves, were all longer in the ATS1 group than the control group. Multivariate logistic regression analysis revealed that BUe could completely differentiate the two groups. PCA ratios in the ATS1 group were significantly larger than the control group (26.5 ± 12.3 vs. 10.4 ± 6.2, *p* = .0005). ICA revealed 1 or 2 U‐wave‐specific independent components (ICs) that exclusively comprise the U wave in ATS1, whereas U waves in the control group were composed of some ICs that also comprised T waves.

**Conclusions:**

U‐wave‐related temporal parameters, particularly BUe, and the existence of U‐wave‐specific ICs, extracted in the ICA, are useful for differentiation of U waves in ATS1 from those in healthy individuals.

## 1 INTRODUCTION

Andersen–Tawil syndrome (ATS), an autosomal‐dominant or sporadic disorder, is characterized by ventricular arrhythmias, periodic paralysis, and dysmorphic facial and skeletal features, although patients with ATS do not necessarily exhibit all these features (Kimura et al., 2012; Plaster et al., 2001). ECG findings of ATS include large U waves, a prolonged repolarization process, frequent premature ventricular contractions (PVCs), and polymorphic/bidirectional ventricular tachycardia (PMVT/BiVT) (Haruna et al., 2007). However, ECG characteristics may overlap with other primary electrical disorders, such as catecholaminergic polymorphic ventricular tachycardia (CPVT) or long QT syndrome (LQTS) (Barajas‐Martinez et al., 2011; Tully et al., 2015). Large U waves may also appear in healthy individuals at low heart rates, although their duration and amplitude fluctuate depending on the status of autonomic nervous system (Magnano, Holleran, Ramakrishnan, Reiffel, & Bloomfield, 2004), making it difficult to confirm the diagnosis of ATS based only on the abovementioned ECG findings. Furthermore, the electrophysiological mechanisms underlying the appearance of the U waves have not been fully clarified. Although ATS has been classified as LQTS type 7, it is the QU intervals rather than QT intervals that are typically prolonged in ATS, leading to some researchers to suggest that ATS should be excluded from the LQTS list (Haruna et al., 2007; Zhang et al., 2005).

Mutations in the *KCNJ2* gene, which encodes the alpha‐subunit of the potassium channel Kir2.1, are identified in about 60% of ATS cases, which are classified as Andersen–Tawil syndrome type 1 (ATS1), and approximately 30% of these are de novo mutations (Nguyen, Pieper, & Wilders, 2013). The Kir2.1 channel functions at the last part of repolarization, and the presence of myocardial cells with loss of function of the channel might distort TU wave complex morphology. However, the mechanisms explaining the formation of large U waves via the mutated channels have not been fully elucidated.

Electrocardiographically, Zhang et al revealed characteristic TU wave complex patterns observed in ATS1, including a prolonged terminal portion of T‐wave downslope, a wide T‐U junction, biphasic U waves, and enlarged U waves (Zhang et al., 2005). Subsequently, Kukla, Biernacka, Baranchuk, Jastrzebski, and Jagodzinska (2014) reported five additional electrocardiographic clues to the diagnosis of ATS1. We hypothesized that the large U waves observed in ATS1 have different morphological and temporal characteristics from those of healthy individuals due to abnormal channel currents, and in this study used multivariate analysis applied to T‐U wave areas of digitized ECG to investigate whether they can be elucidated.

## 2 METHODS

### 2.1 Study population

We studied 13 patients (age 6–69, median 28 years; eight females) with genetically confirmed ATS1 (positive for *KCNJ2* gene mutation) (ATS1 group) and 13 age‐matched healthy individuals free from cardiovascular diseases and medications with electrophysiological effects (control group). Clinical characteristics of the participants in the ATS1 group, including facial dysmorphic features, short stature, periodic paralysis, and ventricular arrhythmias, and mutation types of *KCNJ2* gene are presented in Table 1. Five of the 13 patients were taking antiarrhythmic drugs: two patients took flecainide, one flecainide combined with beta‐blocker, one mexiletine combined with beta‐blocker, and one verapamil (Table 1). Discontinuation of the drugs during the study period was considered risky for these five patients because their ventricular arrhythmias were incessant. The remaining eight patients were not on antiarrhythmic drugs when the ECGs were recorded.

**Table 1:** Clinical characteristics of patients with ATS1

Case no.	Age (years)	Gender	*KCNJ2* genotype	Ventricular arrhythmia	Dysmorphism	Short stature	Periodic paralysis	Medication
1	6	M	R218W	Bidir‐VT	+	+	+	Verap
2	14	F	R67Q	PVC	+	+	−	Carv, Mex
3	17	F	N190I	Bidir‐VT	+	+	−	BB + Flec
4	19	M	R218W	PVC	+	−	+	Flec
5	24	F	R218W	PVC	+	+	+	Flec
6	24	F	R67W	Bidir‐VT	−	−	−	–
7	28	F	R218W	Bidir‐VT	+	+	+ (once)	–
8	34	M	R67W	Bidir‐VT	+	−	+	–
9	46	F	N190I	PVC	+	+	−	–
10	52	F	R67Q	PVC	+	+	−	–
11	54	F	R218W	PVC	+	+	+	–
12	56	M	R218Q	Bidir‐VT	+	−	+	–
13	69	M	R67W	–	−	−	−	–

Abbreviations: BB, beta‐blocker; Bidir‐VT, bidirectional ventricular tachycardia; Carv, carvedilol; Flec, flecainide; Mex, mexiletine; PVC, premature ventricular contraction; Verap, verapamil.

The study protocol was approved by the Ethics Committee of the University of Tsukuba Hospital (Tsukuba, Ibaraki, Japan), and informed consent was obtained from each participant or from parents if the participant was aged <15 years.

### 2.2 Sampling of ECG Data

The methods of ECG data sampling are described in detail in our previous report (Horigome et al., 2011). Briefly, ECGs were recorded using an ECG amplifier (Polymate AP 1532; TEAC) from 10 channels using 20 silver‐chloride surface electrodes. The recorded data were digitized online using an A/D converter (EC‐2360; Elmec) at a sampling rate of 2 kHz.

### 2.3 Measurement of temporal and amplitude parameters of TU wave complex

For each participant, waveforms from the 10 channels exhibiting large U waves (V2 or V3 lead) were selected for analysis. Temporal parameters were measured on raw tracings, or those following first and second‐order differential, and obtained as corrected values using the formula: $\text{divided}\;\text{by}\;\sqrt{\text{RR}}$. Amplitude parameters included T peak (mV) and U peak (mV). All these values were measured after signals averaging 10 beats.

**Figure 1:**
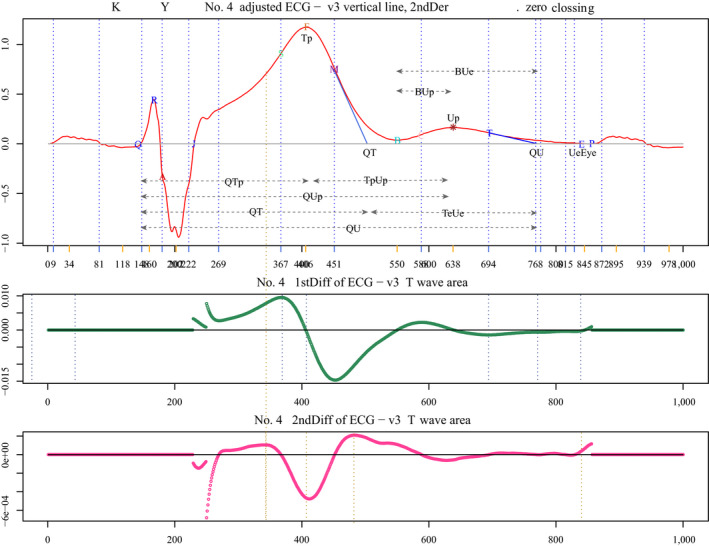
Diagram of typical ECGs. Raw tracing (upper panel), that after first (middle panel), and second‐order differential (lower panel) of ECG. Refer to the text for definitions of each parameter. All temporal parameters are corrected using $\sqrt{\text{RR}}$. T and U ends are the points at which tangents drawn to the steepest down slopes of each wave cross the isoelectric line (tangential method). B (bottom) is the lowest point between the bifid TU complex

The parameters measured (shown in Figure 1) were: time from Q onset to T end/$\sqrt{\text{RR}}$ (QTc) (s); time from Q onset to U end/$\sqrt{\text{RR}}$, (QUc) (s); time from Q onset to T peak (QTp) (s); time from Q onset to U peak (QUp) (s); time from T end to U end (TeUe) (s); time from T peak to U peak (TpUp) (s); time from bottom between T and U (B) to U peak (BUp) (s); time from B to U end (BUe) (s) or U duration; T peak amplitude (Tp) (mV); U peak amplitude, (Up) (mV); U/T amplitude ratio (U/T); where T and U ends are the points at which tangents drawn to the steepest down slopes of each wave crossed the isoelectric line (tangential method), and where bottom was the lowest point between the bifid TU complex.

### 2.4 Principal component analysis (PCA)

Principal component analysis (PCA) was applied to the TU complex segment, from J‐point to U end. The component with the largest variance was extracted as an eigenvector, that perpendicular to the eigenvector was extracted as the second component, that perpendicular to the second component was extracted as the third component, and so on. PCA ratio, a frequently used parameter in PCA, was calculated as the ratio of the second component to the first component of the eigenvector for TU wave complex.

### 2.5 Independent component analysis (ICA) and inverse ICA (i‐ICA)

The methods used for and independent component analysis (ICA) and inverse ICA (i‐ICA) are described in detail in our previous report (Horigome et al., 2013, 2011). Briefly, they involve the following four main steps:

1. noise reduction by wavelet thresholding method;
2. radical ICA with additive random noise;
3. selection of the best model from the results of repeated ICAs; and
4. estimation of origin of each IC on the observed surface ECG (i‐ICA).

In the present study, the number of ICs that comprise the TU complex and the origins of the ICs that comprise the U wave were determined.

### 2.6 Statistical analysis

Continuous variables are expressed as mean ± *SD*, and Student's *t* test was used for comparison. Multivariate logistic regression model selection was then carried out in the bestglm package of the R package to find the best fit using Akaike's information criterion (AIC) (McLeod & Xu, 2018). Logistic regression is a method for fitting a regression curve, *y* = *f*(*x*), where *y* is a categorical variable (ATS1 = 0 or Normal = 1 in this study). This model was used to predict *y* given a set of predictors (all explanatory variables were related to U wave in this study). *p* values of <.05 were considered statistically significant. A receiver operating characteristic (ROC) curve analysis was also performed for each parameter, yielding both an estimate of the area under the curve (AUC) and cutoff values that could be used for the prediction of ATS1. Data were analyzed using R package.

## 3 RESULTS

### 3.1 Temporal parameters

Although QUc and QUp were longer in the ATS1 group than the control group, QTc and QTp were comparable between the two groups. TeUe, TpUp, BUp, and BUe (U duration) were all significantly longer in the ATS1 group than in the control group (Table 2). For multivariate logistic regression analysis, the following 15 explanatory variables were used: Tp, Tpy, Te, B, Up, Upy, Ue, Uey, UeTe, JTe, BUp, BUe, UpUe, TpUp, and PCA ratio. Since the family is non‐Gaussian, Morgan‐Tatar search was used for best subset selection. Best fit may be found using the AIC.

**Table 2:** Comparison of parameters between patients with ATS1 and healthy individuals (control)

	ATS1 (*n* = 13)	Control (*n* = 13)	*p* value
HR (bpm)	62.8 ± 11.5	63.8 ± 7.5	NS
QTc (s)	0.400 ± 0.031	0.388 ± 0.017	NS
QUc (s)	0.670 ± 0.033	0.599 ± 0.026	<.0001
QTp (s)	0.289 ± 0.018	0.301 ± 0.020	NS
QUp (s)	0.538 ± 0.025	0.497 ± 0.023	.0002
TeUe (s)	0.269 ± 0.029	0.211 ± 0.023	<.0001
TpUp (s)	0.249 ± 0.019	0.196 ± 0.021	<.0001
BUp (s)	0.101 ± 0.013	0.063 ± 0.013	<.0001
BUe (s)	0.232 ± 0.018	0.165 ± 0.017	<.0001
Up amplitude (mV)	0.166 ± 0.063	0.062 ± 0.033	<.0001
Tp amplitude (mV)	0.456 ± 0.261	0.767 ± 0.326	.0132
U/T ratio	0.465 ± 0.387	0.0895 ± 0.0557	<.0001
PCA ratio	26.5 ± 12.3	10.4 ± 6.2	.0005
number of T‐ICs	4 (*n* = 13)	4 (*n* = 13)	NA
number of U‐specific ICs	2 (*n* = 11), 1 (*n* = 2)	0 (*n* = 13)	NA

Note

Refer to the text for abbreviations and definitions of each parameter. All temporal parameters are corrected by $\sqrt{\text{RR}}$.

Abbreviations: ATS1, Andersen–Tawil syndrome type 1; HR, heart rate; IC, independent component; NA, not applicable; NS, not significant; PCA, principal component analysis.

*y*(BUe) = exp(35.89 + 37.89 × BUe)/(1 + exp(35.89 + 37.89 × BUe)) was adopted as the best model (AIC = 4). With this equation, ATS and normal could be completely separated with no overlaps.

### 3.2 Amplitude parameters

Although U peak amplitude was larger in ATS1 than the control group, T peak amplitude was inversely larger in the control group than the ATS1 group. These results made the U/T amplitude ratio much larger in the ATS1 group than in the control group (Table 2).

### 3.3 PCA

Principal component analysis ratio in the ATS1 group was significantly larger than that in the control group (26.53 ± 12.33% vs. 10.39 ± 6.24%, *p* = .00054), indicating dyssynchronous repolarization process in ATS1 (Table 2).

### 3.4 ICA

**Figure 2:**
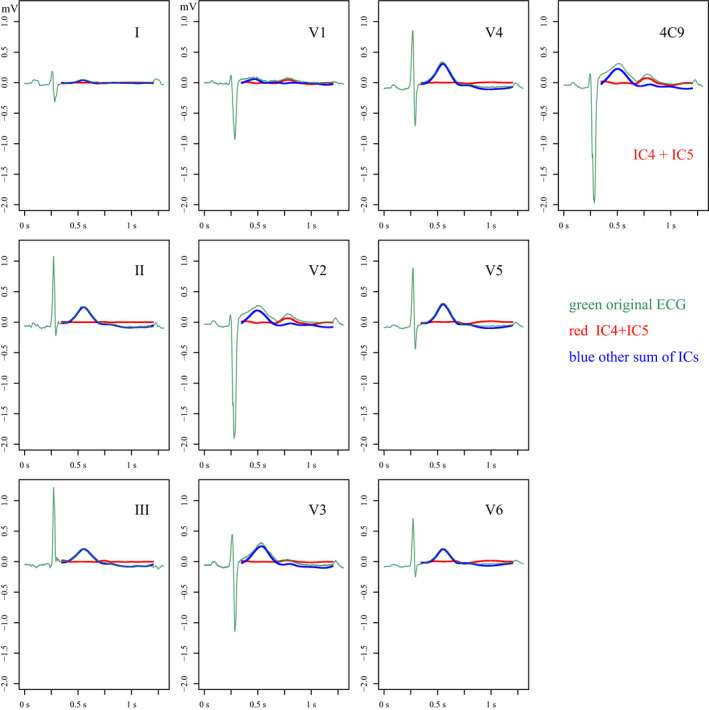
Results of independent component analysis (ICA) and inverse ICA (i‐ICA) in a patient with Andersen–Tawil syndrome type 1 (ATS1). The number of ICs was 10 because ECG data were obtained with 10 leads, and 6 of those constituted the TU wave complex. The 10 ICs were numbered in order of appearance not on the T wave but by the results of the ICA. In this patient with ATS1, the TU wave included 6 ICs. Two of the 6 ICs were added (IC4 + IC5) and are represented by red lines. The remaining 4 ICs were added and are represented by blue lines. The results of i‐ICA are also shown. The green waveforms represent the original ECG, and the red waveforms represent the distribution of IC4 + IC5 on the original leads, making it possible to recognize the origin of IC4 + IC5 on the original ECG. IC4 + IC5 exclusively comprise the U wave without contribution to formation of the T wave, typically shown in leads V2–V3 and 4C9. In all 13 patients with ATS1, 1 or 2 of this kind of U‐wave‐specific ICs were extracted

**Figure 3:**
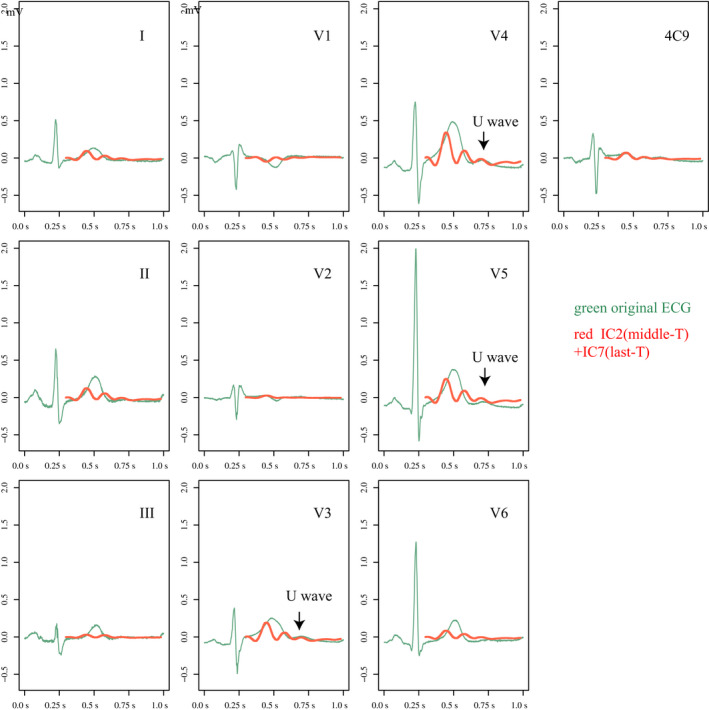
Results of independent component analysis (ICA) and inverse ICA (i‐ICA) in a healthy individual. The number of ICs was 10 because ECG data were obtained with 10 leads, and 4 of them constituted the normal TU wave complex. In all 13 healthy individuals (control group), the TU wave included 4 ICs. Two of the 4 ICs were added (IC2 + IC7) and are represented by red lines. The results of i‐ICA are also shown. The green waveforms represent the original ECG, and the red waveforms represent the distribution of IC2 + IC7 on the original leads, making it possible to recognize the origin of IC2 + IC7 on the original ECG. The U wave was composed of some of the ICs constituting the T wave, typically shown in leads V3–V5. In all 13 healthy individuals of the control group, the U wave was formed by some of ICs for T waves, and no U‐wave‐specific IC were extracted

Independent component analysis revealed that the number of ICs that compose the T wave was 4 in both the ATS1 group and the control group. However, i‐ICA showed that in the ATS1 group the ICs comprising the U wave did not form any part of the T wave but exclusively formed the U wave, whereas all U waves in the control group were composed of some ICs for the T wave. The number of U wave‐specific ICs in ATS1 was 1 (*n* = 2) or 2 (*n* = 11), making the number of ICs for TU wave complex 5 or 6 in ATS1 and 4 in controls. Examples of i‐ICA for a patient with ATS1 and a healthy individual (control) are shown in Figures 2 and 3.

### 3.5 ROC curve analysis

Area under the curve, sensitivity, specificity, and cutoff values for each parameter are shown in Table 3. The AUC values of U‐wave‐related parameters (QUc, TeUe, TpUp, BUp, BUe, and U amp) were >0.9 (indicating high accuracy). Particularly, the AUC of BUe was 1.0, coincident with the result of our multivariate logistic regression analysis that BUe (U duration) could completely differentiate U waves of the ATS1 group from those of the control group.

**Table 3:** Results of ROC curve analysis

Parameter	AUC	Sensitivity	Specificity	Cutoff value
QTc	0.598	5/13	13/13	0.41 (s)
QUc	0.941	12/13	11/13	0.62 (s)
QTp	0.314	2/13	11/13	0.32 (s)
QUp	0.882	11/13	12/13	0.53 (s)
TeUe	0.959	12/13	12/13	0.24 (s)
TpUp	0.976	12/13	12/13	0.27 (s)
BUe	1	13/13	13/13	0.21 (s)
BUp	0.988	12/13	13/13	0.09 (s)
T amplitude	0.805	10/13	11/13	0.59 (s)
U amplitude	0.941	12/13	11/13	0.09 (s)
PCA ratio	0.875	10/13	11/13	17.5 (%)

Note

Refer to the text for abbreviations and definitions of each parameter. All temporal parameters are corrected using $\sqrt{\text{RR}}$.

Abbreviations: AUC, area under curve; PCA, principal component analysis.

## 4 DISCUSSION

In the present study, we analyzed the TU wave complex on ECGs recorded with a high frequency (2 kHz) sampling system in patients with ATS1, and the results were compared with those of healthy individuals. Among the temporal parameters measured, QUc, QUp, TeUe, TpUp, BUp, and BUe (U‐wave duration) were longer in the ATS1 group than in the control group, although QTc and QTp were both comparable between the two groups. It is worth noting that there were no overlaps in the values of BUe (U duration) between the two groups, and multivariate logistic regression analysis revealed that this parameter is the best model for prediction of U waves of ATS1. As for amplitude parameters, U‐wave amplitude was higher, and T‐wave amplitude was lower in the ATS1 group than in the control group, making the U/T ratio larger in ATS1. Similar results have been previously reported. Zhang et al described characteristic ECG phenotypes of ATS1, including prolonged terminal T downslope, wide T‐U junction, and biphasic and enlarged U waves. U‐wave duration and amplitude were significantly larger than those in ATS patients without *KCNJ2* mutation or healthy individuals, just as observed in the present study. They also showed that not QTc but QUc was increased in ATS1 compared with patients without KCNJ2 mutation and healthy individuals (Zhang et al., 2005). Recently, Kukla et al. (2014) reported five additional electrocardiographic clues to the diagnosis of ATS1, two of which being related to large U waves.

Andersen‐Tawil syndrome has been classified as long QT syndrome (LQTS) type 7 based on prolonged QT intervals due to mutated ion channel protein, leading to ventricular arrhythmias (Tristani‐Firouzi et al., 2002). However, QTc and QTp in ATS1 were both only slightly prolonged or comparable with the control group in our study as it was in previous studies; whereas QUc and QUp were both significantly longer in the ATS1 group than the control group (Zhang et al., 2005). These results suggest that the typical repolarization abnormality in ATS1 is not QT prolongation but rather QU prolongation, and this syndrome should be annotated as ATS with *KCNJ2* mutation rather than being listed as LQTS type 7.

In the present study, we demonstrated, using ROC curve analysis, that several U‐wave‐related parameters (QUc, TeUe, TpUp, BUp, BUe, and U amp) were useful for prediction of U waves of ATS1 as these had a high AUC values of >0.9. Their cutoff values are presented in Table 3.

We also tried to characterize the TU wave complex by multivariate statistical methods, that is, PCA and ICA. In PCA, we used the PCA ratio (the ratio of the second component to the first component of the eigenvector) as a parameter for representation of dyssynchrony in myocardial repolarization. PCA ratio has already been shown to be a useful diagnostic tool for the diagnosis of myocardial dyssynchrony in LQTS as the PCA ratio in LQTS is higher than that in healthy individuals (31.6 ± 21.3% vs. 16.8 ± 8.5% in our previous study) (Horigome et al., 2013; Priori et al., 1997). Okin et al demonstrated that the PCA ratio of the T wave was useful for prediction of cardiovascular mortality (Okin et al., 2002). PCA ratios in the ATS1 group in this study (26.5 ± 12.3%) were significantly higher than those in the control group; however, whether this is effective for differentiation of ATS1 from the other types of LQTS remains to be investigated.

Independent component analysis is a multivariate statistical method that can be used to extract source signals from the observed signals under the assumption that an observed signal is a linear mixture of non‐Gaussian source components, which are independent of one another. In a previous study, we applied ICA to T waves on digitized high frequency ECG data and showed that normal T waves consist exclusively of four ICs, whereas those of LQT type 1 consist of five or more ICs, including additional ICs (Horigome et al., 2011). In the present study, the number of ICs that comprise T waves was 4 in healthy controls, the same number as in our previous study. However, 1 or 2 additional ICs were extracted in all participants with ATS1 when TU wave complex was analyzed using ICA. i‐ICA revealed that these additional ICs exclusively comprised U waves without contributing to the formation of T waves on the original ECG (Figure 2). U waves in control group included some ICs that were not U‐wave‐specific but were mainly involved in formation of T waves (Figure 3).

While the exact origin of the large U waves observed in ATS1 still remains unknown, one possible mechanism is related to intrinsic potential differences at the terminal points of action potentials, resulting in spatial dispersion of repolarization in which some parts of the ventricles, such as Purkinje fibers and papillary muscles, have a longer repolarization process (Postema et al., 2009). We studied patients with ATS1 (positive for *KCNJ2* mutation), which is identified in approximately 60% of genotype‐proved ATS (Donaldson et al., 2003). *KCNJ2* encodes Kir2.1 channel, which plays a critical role in maintaining the stable membrane potential through the inward rectifier potassium current, *IK1*, and contributes to the terminal phase of repolarization (Lopatin & Nichols, 2001). *KCNJ2* mutation‐induced reduction of *Ik1* could augment the U wave through several mechanisms. As spatial dispersion of Kir2.1 is a possible mechanism of U‐wave formation in healthy individuals (Watanabe, 1975), existence of mutated channels can further increase spatial heterogeneity of the terminal phase of repolarization. Lengthening of action potential duration via pharmacological *Ik1* block was also demonstrated in animal models. Morita et al, using a canine tissue model of ATS1 created with cesium chloride (a suppressor of *Ik1*), demonstrated that delayed late phase 3 repolarization of action potential and delayed after depolarization were both related to the appearance of large U waves (Morita, Zipes, Morita, & Wu, 2007). This enhanced heterogeneity could account for the appearance of U‐wave‐specific ICs as extracted with ICA that reflects delayed repolarization area. However, it should be noted that specific ion channels or confined myocardial areas do not necessarily correspond individually to particular ICs. Also, if the U wave originates from delayed Purkinje fiber or ventricular myocyte repolarization, it might appear as a prolonged or notched T wave rather than as a separate deflection or a U‐wave‐specific IC (Zhang et al., 2005).

## 5 STUDY LIMITATIONS

Limitations of the present study include the small number of patients with ATS1, of whom some were receiving antiarrhythmic agents during the study period, which might have affected the results. However, discontinuation of the drugs was considered risky as they had constant ventricular arrhythmias, and we waited for appearance of sinus beats even short in duration. Such medication has reportedly less effects on U waves (Miyamoto et al., 2015), and actually large U waves were apparent in these cases. We also compared the values of U‐wave‐related parameters, including QUc and BUe, between ATS1 patients with medication and those without and found no differences in the values (data are not shown in Section 3).

## 6 CONCLUSIONS

This study indicates that U waves in patients with ATS1 can be differentiated from those in healthy individuals by several parameters of the TU wave complex, especially U‐wave‐related temporal parameters. Furthermore, ICA extracted U‐wave‐specific ICs that exclusively comprise U waves in ATS1 are also useful for the diagnosis of the disease, although the mechanisms of independency of the ICs from T wave remain to be clarified.

## CONFLICT OF INTEREST

There is no conflict of interest.

## References

[anec12721-bib-0001] Barajas‐Martinez, H. , Hu, D. , Ontiveros, G. , Caceres, G. , Desai, M. , Burashnikov, E. , … Antzelevitch, C. (2011). Biophysical and molecular characterization of a novel de novo KCNJ2 mutation associated with Andersen‐Tawil syndrome and catecholaminergic polymorphic ventricular tachycardia mimicry. Circulation. Cardiovascular Genetics, 4(1), 51–57. 10.1161/CIRCGENETICS.110.957696 21148745 PMC3041844

[anec12721-bib-0002] Donaldson, M. R. , Jensen, J. L. , Tristani‐Firouzi, M. , Tawil, R. , Bendahhou, S. , Suarez, W. A. , … Ptacek, L. J. (2003). PIP2 binding residues of Kir2.1 are common targets of mutations causing Andersen syndrome. Neurology, 60(11), 1811–1816. 10.1212/01.wnl.0000072261.14060.47 12796536

[anec12721-bib-0003] Haruna, Y. , Kobori, A. , Makiyama, T. , Yoshida, H. , Akao, M. , Doi, T. , … Horie, M. (2007). Genotype‐phenotype correlations of KCNJ2 mutations in Japanese patients with Andersen‐Tawil syndrome. Human Mutation, 28(2), 208 10.1002/humu.9483 17221872

[anec12721-bib-0004] Horigome, H. , Ishikawa, Y. , Kato, Y. , Nakamura, A. , Iwamoto, M. , Sumitomo, N. , & Yoshinaga, M. (2013). Analysis of T wave in congenital long QT syndrome by independent component analysis: Comparison of its diagnostic accuracy with principal component analysis. Journal of Cardiology – Japanese Edition, 8(1), 14–25.

[anec12721-bib-0005] Horigome, H. , Ishikawa, Y. , Shiono, J. , Iwamoto, M. , Sumitomo, N. , & Yoshinaga, M. (2011). Detection of extra components of T wave by independent component analysis in congenital long‐QT syndrome. Circulation. Arrhythmia and Electrophysiology, 4(4), 456–464. 10.1161/CIRCEP.110.958827 21511995

[anec12721-bib-0006] Kimura, H. , Zhou, J. , Kawamura, M. , Itoh, H. , Mizusawa, Y. , Ding, W. G. , … Horie, M. (2012). Phenotype variability in patients carrying KCNJ2 mutations. Circulation. Cardiovascular Genetics, 5(3), 344–353. 10.1161/CIRCGENETICS.111.962316 22589293

[anec12721-bib-0007] Kukla, P. , Biernacka, E. K. , Baranchuk, A. , Jastrzebski, M. , & Jagodzinska, M. (2014). Electrocardiogram in Andersen‐Tawil syndrome. New electrocardiographic criteria for diagnosis of type‐1 Andersen‐Tawil syndrome. Current Cardiology Reviews, 10(3), 222–228. 10.2174/1573403X10666140514102528 24827800 PMC4040873

[anec12721-bib-0008] Lopatin, A. N. , & Nichols, C. G. (2001). Inward rectifiers in the heart: An update on I(K1). Journal of Molecular and Cellular Cardiology, 33(4), 625–638. 10.1006/jmcc.2001.1344 11273717

[anec12721-bib-0009] Magnano, A. R. , Holleran, S. , Ramakrishnan, R. , Reiffel, J. A. , & Bloomfield, D. M. (2004). Autonomic modulation of the u wave during sympathomimetic stimulation and vagal inhibition in normal individuals. Pacing and Clinical Electrophysiology, 27(11), 1484–1492. 10.1111/j.1540-8159.2004.00665.x 15546302

[anec12721-bib-0010] McLeod, A. I. , & Xu, C. (2018). “bestglm”: Best Subset GLM and Regression Utilities. R package version 0.37. Retrieved from https://CRAN.R-project.org/package=bestglm

[anec12721-bib-0011] Miyamoto, K. , Aiba, T. , Kimura, H. , Hayashi, H. , Ohno, S. , Yasuoka, C. , … Shimizu, W. (2015). Efficacy and safety of flecainide for ventricular arrhythmias in patients with Andersen‐Tawil syndrome with KCNJ2 mutations. Heart Rhythm: the Official Journal of the Heart Rhythm Society, 12(3), 596–603. 10.1016/j.hrthm.2014.12.009 25496985

[anec12721-bib-0012] Morita, H. , Zipes, D. P. , Morita, S. T. , & Wu, J. (2007). Mechanism of U wave and polymorphic ventricular tachycardia in a canine tissue model of Andersen‐Tawil syndrome. Cardiovascular Research, 75(3), 510–518. 10.1016/j.cardiores.2007.04.028 17531215

[anec12721-bib-0013] Nguyen, H. L. , Pieper, G. H. , & Wilders, R. (2013). Andersen‐Tawil syndrome: Clinical and molecular aspects. International Journal of Cardiology, 170(1), 1–16. 10.1016/j.ijcard.2013.10.010 24383070

[anec12721-bib-0014] Okin, P. M. , Devereux, R. B. , Fabsitz, R. R. , Lee, E. T. , Galloway, J. M. , & Howard, B. V. (2002). Principal component analysis of the T wave and prediction of cardiovascular mortality in American Indians: The Strong Heart Study. Circulation, 105(6), 714–719. 10.1161/hc0602.103585 11839627

[anec12721-bib-0015] Plaster, N. M. , Tawil, R. , Tristani‐Firouzi, M. , Canún, S. , Bendahhou, Saïd , Tsunoda, A. , … Ptáček, L. J. (2001). Mutations in Kir2.1 cause the developmental and episodic electrical phenotypes of Andersen's syndrome. Cell, 105(4), 511–519. 10.1016/s0092-8674(01)00342-7 11371347

[anec12721-bib-0016] Postema, P. G. , Ritsema van Eck, H. J. , Opthof, T. , van Herpen, G. , van Dessel, P. F. H. M. , Priori, S. G. , … Wilde, A. A. M. (2009). IK1 modulates the U‐wave: Insights in a 100‐year‐old enigma. Heart Rhythm: the Official Journal of the Heart Rhythm Society, 6(3), 393–400. 10.1016/j.hrthm.2008.11.024 19251218

[anec12721-bib-0017] Priori, S. G. , Mortara, D. W. , Napolitano, C. , Diehl, L. , Paganini, V. , Cantù, F. , … Schwartz, P. J. (1997). Evaluation of the spatial aspects of T‐wave complexity in the long‐QT syndrome. Circulation, 96(9), 3006–3012. 10.1161/01.cir.96.9.3006 9386169

[anec12721-bib-0018] Tristani‐Firouzi, M. , Jensen, J. L. , Donaldson, M. R. , Sansone, V. , Meola, G. , Hahn, A. , … Tawil, R. (2002). Functional and clinical characterization of KCNJ2 mutations associated with LQT7 (Andersen syndrome). The Journal of Clinical Investigation, 110(3), 381–388. 10.1172/JCI15183 12163457 PMC151085

[anec12721-bib-0019] Tully, I. , Atherton, J. , Hunt, L. , Ingles, J. , Semsarian, C. , & McGaughran, J. (2015). Rarity and phenotypic heterogeneity provide challenges in the diagnosis of Andersen‐Tawil syndrome: Two cases presenting with ECGs mimicking catecholaminergic polymorphic ventricular tachycardia (CPVT). International Journal of Cardiology, 201, 473–475. 10.1016/j.ijcard.2015.07.069 26322597

[anec12721-bib-0020] Watanabe, Y. (1975). Purkinje repolarization as a possible cause of the U wave in the electrocardiogram. Circulation, 51(6), 1030–1037. 10.1161/01.cir.51.6.1030 1132093

[anec12721-bib-0021] Zhang, L. , Benson, D. W. , Tristani‐Firouzi, M. , Ptacek, L. J. , Tawil, R. , Schwartz, P. J. , … Vincent, G. M. (2005). Electrocardiographic features in Andersen‐Tawil syndrome patients with KCNJ2 mutations: Characteristic T‐U‐wave patterns predict the KCNJ2 genotype. Circulation, 111(21), 2720–2726. 10.1161/CIRCULATIONAHA.104.472498 15911703

